# PINK1-Associated Parkinson's Disease Is Caused by Neuronal Vulnerability to Calcium-Induced Cell Death

**DOI:** 10.1016/j.molcel.2009.02.013

**Published:** 2009-03-13

**Authors:** Sonia Gandhi, Alison Wood-Kaczmar, Zhi Yao, Helene Plun-Favreau, Emma Deas, Kristina Klupsch, Julian Downward, David S. Latchman, Sarah J. Tabrizi, Nicholas W. Wood, Michael R. Duchen, Andrey Y. Abramov

**Affiliations:** 1 Department of Molecular Neuroscience, Institute of Neurology, Queen Square, London WC1N 3BG, UK; 2 Cancer Research UK, 44 Lincoln's Inn Fields, London WC2A 3PX, UK; 3 Medical Molecular Biology Unit, Institute of Child Health, 30 Guilford Street, London WC1N 1EH, UK; 4 Department of Physiology, University College London, London WC1E 6BT, UK; 5 Birkbeck, University of London, Malet Street, London WC1E 7HX, UK; 6 Department of Neurodegenerative Disease, Institute of Neurology, Queen Square, London WC1N 3BG, UK

**Keywords:** CELLBIO, CELLCYCLE

## Abstract

Mutations in *PINK1* cause autosomal recessive Parkinson's disease. PINK1 is a mitochondrial kinase of unknown function. We investigated calcium homeostasis and mitochondrial function in PINK1-deficient mammalian neurons. We demonstrate physiologically that PINK1 regulates calcium efflux from the mitochondria via the mitochondrial Na^+^/Ca^2+^ exchanger. PINK1 deficiency causes mitochondrial accumulation of calcium, resulting in mitochondrial calcium overload. We show that calcium overload stimulates reactive oxygen species (ROS) production via NADPH oxidase. ROS production inhibits the glucose transporter, reducing substrate delivery and causing impaired respiration. We demonstrate that impaired respiration may be restored by provision of mitochondrial complex I and II substrates. Taken together, reduced mitochondrial calcium capacity and increased ROS lower the threshold of opening of the mitochondrial permeability transition pore (mPTP) such that physiological calcium stimuli become sufficient to induce mPTP opening in PINK1-deficient cells. Our findings propose a mechanism by which PINK1 dysfunction renders neurons vulnerable to cell death.

## Introduction

Mitochondrial dysfunction has been implicated in a range of neurodegenerative diseases, in particular, Parkinson's disease (PD). PD is a common neurodegenerative disease characterized initially by loss of dopaminergic neurons in the substantia nigra (SN), and later, by more widespread nondopaminergic neuronal loss (Braak et al., 2003). The molecular pathogenesis of sporadic PD, and the basis for selective dopaminergic neuronal loss, remains unclear. Several genes have now been identified in which mutations cause forms of familial PD that are clinically and pathologically indistinguishable from sporadic PD. A number of these genes, namely *PINK1*, *DJ-I*, and *Omi/HtrA2*, encode mitochondrially located proteins, further implicating mitochondrial dysfunction as the primary event sufficient to cause PD.

Mutations in the PINK1 gene cause autosomal recessive PD (Valente et al., 2004). PINK1 is a 581 amino acid protein consisting of a mitochondrial targeting motif and a serine/threonine kinase domain homologous to the Ca^2+^/calmodulin family. Studies both in vitro (Silvestri et al., 2005; Muqit et al., 2006) and in vivo (Gandhi et al., 2006) show PINK1 localized to mitochondria. The targets for PINK1 remain unknown, although mitochondrial proteins such as Omi/HtrA2 (Plun-Favreau et al., 2007) and TRAP1 (Pridgeon et al., 2007) have been identified as downstream effectors of PINK1 function. It is well established that PINK1 protects neurons from oxidative stress, and furthermore plays a role in mitochondrial morphology in mammalian cells and *Drosophila* models (Haque et al., 2008; Exner et al., 2007; Yang et al., 2006).

We previously demonstrated that PINK1 deficiency results in an age-related loss of neuronal viability, and increased sensitivity to stress-induced mitochondrial apoptosis. In keeping with other reports, mitochondrial dysfunction was implicated due to the presence of lowered mitochondrial membrane potential, increased oxidative stress, and morphological changes of mitochondria associated with PINK1 deficiency (Wood-Kaczmar et al., 2008). However, the mechanisms underlying the mitochondrial membrane potential, the sources of oxidative stress, and basis of the sensitivity to apoptosis remained unknown. In this study we used dynamic imaging techniques to explore the mitochondrial pathophysiology of PINK1-induced PD. We report that loss of PINK1 function causes dysregulation of mitochondrial calcium handling, resulting in mitochondrial calcium overload which sensitizes the mitochondria to opening of the permeability transition pore (PTP). Furthermore, we explain the basis for the impaired respiration and oxidative stress in PINK1 models of PD. These findings define a mechanism whereby PINK1 dysfunction may cause nigral neuronal death, and highlight pathophysiological processes that may also occur in sporadic PD.

## Results

**Figure 1:**
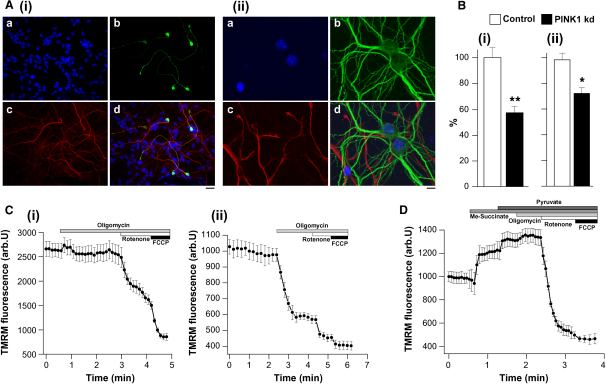
Characteristics of Mitochondrial Membrane Potential in PINK1 KD Neurons (A) Immunofluorescence of human dopaminergic neurons (Ai) (blue, Hoechst; red, β3 tubulin; green, TH) and primary mouse neurons (Aii) (blue, Hoechst; red, GFAP; green, MAP). Scale bar, 10 μM. (B) PINK1 KD neuroblastoma cells showed a 43% reduction (n > 120, p < 0.0001) in basal mitochondrial membrane potential (Δψm) compared to control cells (Bi). PINK1 KD human neurons exhibited 16% reduction (n > 60, p < 0.005) in basal Δψm compared to controls (Bii). (C) In WT mouse neurons (Ci), oligomycin did not affect Δψm; rotenone induced partial depolarization; FCCP induced complete depolarization. In PINK1 KO mouse neurons (Cii), oligomycin induced mitochondrial depolarization (78.6% ± 4.8% decrease in Δψm, n = 72). (D) Application of methyl succinate to PINK1 KO mouse neurons increased basal Δψm (19.5% ± 2.1%); application of pyruvate or malate to PINK1 KO neurons also increased the basal Δψm (18.6% ± 0.7%, n = 87). Substrate provision abolished oligomycin-induced mitochondrial depolarization in PINK1 KO. Error bars represent mean ± SEM.

Using stable expression of shRNA constructs, we were able to reduce PINK1 gene expression by >90% in (1) a human dopaminergic neuroblastoma cell line (SH-SY5Y), and (2) human neurons derived from fetal mesencephalic stem cells that represent the closest in vitro model of primary human neurons. Finally, we corroborated our findings with another mammalian model: (3) primary cortical and midbrain neurons from a PINK1 knockout (KO) transgenic mouse (Figures 1Ai and 1Aii). The characterization of these models has been extensively described (Wood-Kaczmar et al., 2008). Stable re-expression of wild-type (WT)-PINK1 or the kinase-dead mutant K219M-PINK1 was achieved in PINK1 knockdown (KD) neuroblastoma cells and confirmed by RT-PCR.

### Mitochondrial Membrane Potential

Mitochondrial membrane potential (Δψ_m_) is an indicator of mitochondrial state. To control for the effect of multidrug resistance pumps on loading of dyes and experimental results, cells were initially loaded in the presence of the MDR inhibitor cyclosporine H. No difference in loading or in results was detected, and therefore all further experiments were conducted without cyclosporine H.

PINK1 KD in neuroblastoma cells is associated with a significant reduction in the TMRM signal by 43% compared to controls (n > 120 cells in >2 clones, p < 0.0001). Re-expression of WT PINK1, but not K219M-PINK1, rescued the TMRM signal in neuroblastoma cells (data not shown). PINK1 KD in human neurons is also associated with a significant lowering of the mean TMRM fluorescence signal by 16% (n = 60 in two clones, p < 0.005) (Figures 1Bi and 1Bii). These data suggest that PINK1 KD is associated with lowering of the basal Δψ_m._ As basal accumulation of TMRM may be dependent on several factors including plasma membrane potential, we investigated the mechanism maintaining the Δψ_m_ by studying the sensitivity of Δψm to a range of mitochondrial inhibitors.

Control cells demonstrated either no response or slight hyperpolarization in response to complex V inhibition by oligomycin (0.2 μg/ml), while subsequent inhibition of complex 1 by rotenone (5 μM) caused a rapid loss of potential (Figure 1Ci). Therefore, in control neurons, the Δψ_m_ is maintained by the activity of the respiratory chain, with contributions from both complex I and complex II (100% = basal Δψm, 0 = FCCP Δψm at end). In contrast, oligomycin caused marked mitochondrial depolarization in PINK1 KD/KO neurons (78.6% ± 4.8% decrease in TMRM signal in mouse KO cortical neurons, n = 72 cells; 84.6% ± 3.2% for human KD neurons, n = 112 cells; Figure 1Cii). Therefore, in PINK1 KD/KO cells, Δψ_m_ is largely maintained by the hydrolysis of ATP by complex V, rather than by respiration.

Provision of additional substrate for complex 1 (5 mM pyruvate and 5 mM malate) to PINK1 KD neurons caused an increase in Δψ_m_ (TMRM signal increased by 23.4% ± 1.9%, n = 68 for human neurons and by 18.6% ± 0.7%, n = 87 for mouse KO neurons). The membrane-permeable succinate analog (5 mM methyl succinate) also increased basal Δψ_m_ in PINK1 KO neurons (by 19.5% ± 2.1%; Figure 1D). Importantly, the provision of complex I and II substrates to PINK1 KD/KO neurons completely prevented the oligomycin-induced depolarization (Figure 1D).

Thus, respiration is insufficient to maintain the Δψ_m_ in PINK1 KD/KO cells, although the normal mechanisms that maintain Δψ_m_ may be restored in PINK1 KD neurons by increasing substrate supply to complex I and II.

### Respiratory Function

#### Redox State

**Figure 2:**
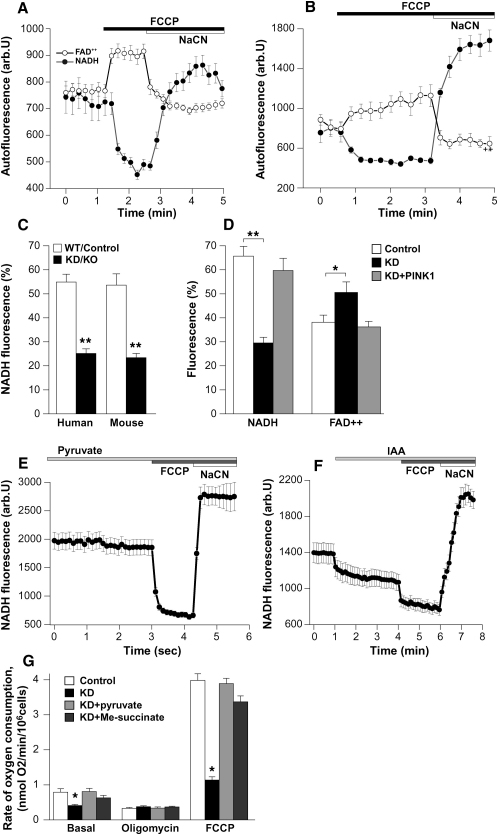
Redox State and Rate of Oxygen Consumption in PINK1 KD/KO and Control Cells (A and B) Graphs demonstrate averaged trace of NADH and FAD^2+^ autofluorescence in neuroblastoma cells ([A], control; [B], PINK1 KD). The response to FCCP (1 μM) is larger in the control cells; the response to cyanide (1 mM) is larger in PINK1 KD cells. (C and D) Quantification of the percentage change in NADH or FAD^2+^ fluorescence: 0 is response to FCCP, and 100% is response to cyanide; this is reversed for FAD fluorescence. PINK1 KD/KO neurons have lower NADH fluorescence compared to control neurons. PINK1 KD neuroblastoma cells have lowered NADH fluorescence and increased FAD^2+^ fluorescence than controls. This can be reversed by re-expression of WT PINK1 in PINK1 KD cells. (E) Preincubation of PINK1 KO mouse neurons with 5 mM pyruvate restored redox level to control values. (F) Preincubation of WT mouse neurons with IAA to inhibit glycolysis reduced redox level to PINK1 KD values. (G) The basal rate of oxygen consumption is reduced in PINK1 KD neuroblastoma cells compared to control cells. PINK1 KD cells show no response to oligomycin. The maximal rate of respiration and oxygen consumption induced by FCCP was significantly lower in PINK1 KD cells than control cells. Pyruvate 5 mM and methyl succinate increased basal oxygen consumption, and increased FCCP induced maximal respiration. Error bars represent mean ± SEM.

The redox state of NADH or FAD^2+^ is a function of respiratory chain activity and the rate of substrate supply. We measured the resting level of NADH and FAD^2+^ autofluorescence and generated the “redox index,” a ratio of the maximally oxidized (response to 1 μM FCCP) and maximally reduced (response to 1 mM NaCN) signals. The redox index of NADH in the PINK1 KD cells was significantly more oxidized than in controls (PINK1 KD neurons, 54.9% ± 3.3%, n = 69, compared to control neurons, 25.2% ± 1.9%, n = 65; PINK1 KD neuroblastoma, 65.7% ± 4.1%, n = 41, compared to control neuroblastoma cells, 29.6% ± 2.3%, n = 39; PINK1 KO mouse neurons, 53.6% ± 4.7% (n = 99), compared to control mouse neurons, 23.4% ± 1.8%, n = 86; p < 0.001 for all type of the cells) (Figure 2). Re-expression of WT, but not kinase mutant PINK1, restored the basal NADH redox level in PINK1 KD neuroblastoma cells to values equivalent to the controls (59.7% ± 5.1%). In addition, FAD^2+^ level was more oxidized in PINK1 KD neuroblastoma cells (PINK1 KD, 49.7% ± 4.3%, compared to control, 37.5% ± 2.9%, p < 0.05; Figures 2A, 2B, and 2D). Re-expression of WT PINK1 recovered FAD^2+^ to control levels (Figure 2D).

The more oxidized redox level in PINK1 KD/KO cells may be due to a rate-limiting substrate supply (Duchen et al., 2003). Preincubation (for 20 min) of PINK1 KO neurons with 5 mM pyruvate restored the redox state to control values (a rise from 23.4% ± 1.8% to 54.8% ± 4.9%; n = 71; p < 0.001) (Figure 2E). In contrast, inhibition of glycolysis by iodoacetic acid (IAA, 10 μM) in WT mouse neurons decreased the redox level to values comparable PINK1 KO neurons (from 53.6% ± 4.7% to 26.1% ± 2.1%; n = 59; p < 0.001) (Figure 2F).

*Oxygen Consumption.* The basal oxygen consumption (Figure 2G) was significantly reduced in the PINK1 KD cells (0.41 ± 0.033 nmol/O_2_/min/10^6^ cells; n = 4 experiments) compared to control cells (0.79 ± 0.101 nmol O_2_/min/10^6^; n = 4 experiments; p < 0.05). Oligomycin 2 μg/ml inhibited the respiration coupled to oxidative phosphorylation in control cells (to 0.33 ± 0.025 nmol O_2_/min/10^6^, p < 0.05), but not in PINK1 KD cells (0.38 ± 0.022 nmol O_2_/min/10^6^ compared to basal 0.41 ± 0.033 nmol/O_2_/min/10^6^). FCCP (1 μM) accelerated respiration to maximal levels in control cells, but to a lesser extent in PINK1 KD cells (3.98 ± 0.19 versus 1.14 ± 0.089 nmol/O_2_/min/10^6^). These data suggest a generalized impairment of respiration in PINK1 KD cells.

Application of 5 mM pyruvate to PINK1 KD neuroblastoma cells resulted in an increase in basal respiration to control values (from 0.41 ± 0.033 nmol/O_2_/min/10^6^ cells to 0.81 ± 0.09 nmol/O_2_/min/10^6^ cells; n = 4 experiments; p < 0.001). Of note, the presence of pyruvate also changed the response of the cells to oligomycin. Maximal respiration induced by FCCP was also significantly higher following addition of pyruvate in PINK1 KD cells (3.89 ± 0.15 versus 1.14 ± 0.089 nmol/O_2_/min/10^6^; p < 0.001; n = 4 experiments). Application of methyl succinate significantly stimulated cell respiration increasing the maximal rate of respiration (basal rate increased to 0.63 ± 0.07 nmol/O_2_/min/10^6^; FCCP-induced respiration increased to 3.37 ± 0.17 nmol/O_2_/min/10^6^; n = 4 experiments; p < 0.001; Figure 2G). Therefore the respiratory chain is intact in PINK1 KD cells and the inhibition of respiration in these cells is due the lack of substrates for complex I.

*Glucose Uptake.* To investigate the availability of substrates for the respiratory chain complexes, we studied the uptake of fluorescent glucose (2-NBDG 2 μM in medium containing low glucose [2 mM]). The rate of glucose uptake in mouse PINK1 KO neurons (cortical and midbrain; n = 114) was significantly slower compared to WT (n = 122) neurons (41.7% ± 3.9% of control; p < 0.001; see Figure S1A available online). In human PINK1 KD neurons, the rate of glucose uptake was 54.1% ± 5.1% of control (n = 58 for control; n = 63 for KD) In PINK1 KD neuroblastoma cells, the rate of glucose uptake was 63.8% ± 5.4% of control cells (n = 112 for control; n = 94 for KD; p < 0.001) (Figure S1B).

PINK1 deficiency is associated with reduced glucose uptake, resulting in a global impairment of respiration. Thus provision of respiratory chain substrates is able to reverse the impaired respiration, the altered redox state and the lowered Δψ_m_.

### Calcium Homeostasis

#### Effect of Physiological and UV-Induced Calcium Stimuli

**Figure 3:**
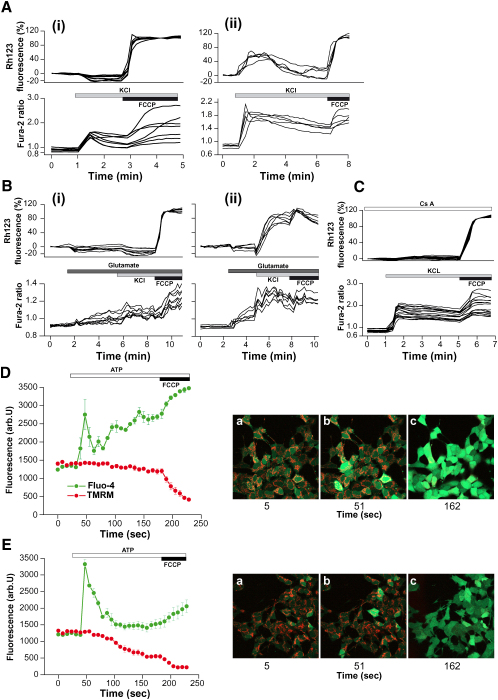
Physiological Calcium Stimuli Induce Mitochondrial Depolarization in PINK1 KD Cells (A–C) Mouse (Ai and Aii) and human (Bi–Biii) neurons were loaded with fura-2 am and Rh123. KCl (50 mM) produced a rise in [Ca^2+^]_c_ in mouse wild-type neurons (Ai) and human control neurons (Bi). In PINK1 KO mouse neurons (Aii) and in PINK1 KD human neurons (Bii), the [Ca^2+^]_c_ was associated with an increase in Rh123 fluorescence and Δψm depolarization. Preincubation of human PINK1 KD neurons with 0.5 μM CsA prevented the Δψm depolarization of cells, but not the [Ca^2+^]_c_ signal (C). (D and E) Human neuroblastoma cells were loaded with fluo-4 (green) and TMRM (red). ATP (100 μM) induced a rise in [Ca^2+^]_c_ and thus an increase in fluo-4 fluorescence. In PINK1 KD cells the ATP-induced [Ca^2+^]_c_ signal was associated with a decrease in TMRM fluorescence and Δψm depolarization. Error bars represent mean ± SEM.

Mitochondria play a major role in maintaining neuronal calcium homeostasis (Szabadkai et al., 2006). In order to examine mitochondrial calcium handling in the PINK1 KD/KO cells, we measured fura-2 and Rh123 fluorescence (dequench mode) simultaneously as indicators of [Ca^2+^]_c_ and Δψ_m_. Application of 50 mM KCl depolarizes the plasma membrane in neurons, induces the opening of potential-sensitive calcium channels, causing a rise in [Ca^2+^]_c_. Application of 50 mM KCl to WT mouse neurons produced a [Ca^2+^]_c_ signal, with no significant mitochondrial depolarization as characterized before (Keelan et al., 1999) (Figure 3Ai). KCl (50 mM) application to PINK1 KO neurons induced a much higher [Ca^2+^]_c_ signal (signal increase: control 0.52 ± 0.001, n = 134, compared to 1.12 ± 0.1 in PINK1 KD, n = 187; p < 0.001; Figure 3Aii), which was associated with a major loss of Δψ_m_ (the Rh123 signal rose by 68.9% ± 5.7%, n = 187) (Figure 3Aii). In human neurons, KCl induced a small rise in the [Ca^2+^]_c_ in control neurons (Figure 3Bi) and a much higher [Ca^2+^]_c_ in PINK1 KD neurons (0.36 ± 0.1, n = 133 in control compared to 0.76 ± 0.41, n = 102; Figure 3Bii). The higher [Ca^2+^]_c_ in PINK1 KD neurons was associated with profound lowering of Δψ_m_ (Rh123 signal increased by 94% ± 6.6%). The mitochondrial depolarization was prevented by preincubation with the mPTP inhibitor cyclosporin A (CsA) (0.5 μM; Figure 3C).

Neuroblastoma cells were loaded with fluo-4 and TMRM (redistribution mode) for simultaneous measurement of [Ca^2+^]_c_ and Δψm. Application of 100 μM ATP to neuroblastoma cells induced a [Ca^2+^]_c_ signal in both control (n = 61; Figure 3D) and PINK1 KD cells (n = 56; Figure 3E). The [Ca^2+^]_c_ signal in the PINK1 KD cells was again significantly larger than in control cells and again induced a mitochondrial depolarization (TMRM signal decreased by 65.7 ± 5.8%, n = 56). Calcium-induced depolarization in PINK1 KD neuroblastoma cells was prevented by preincubation of cells with 0.5 μM CsA (n = 48; data not shown).

**Figure 4:**
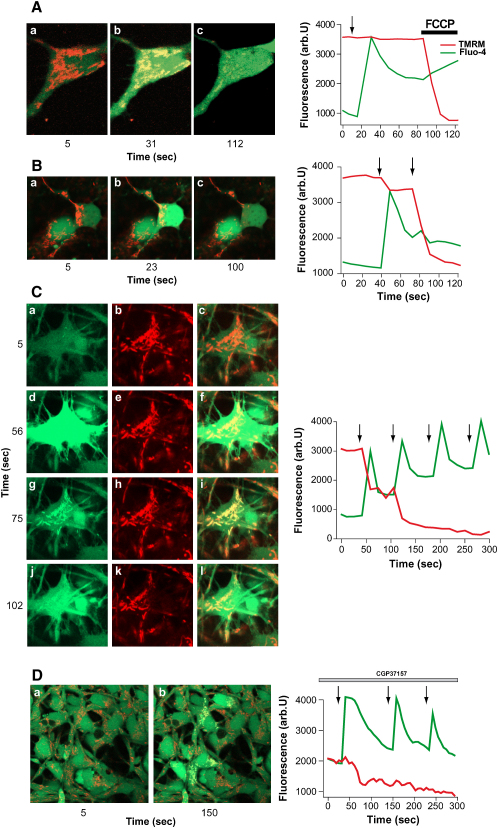
A Rise in [Ca^2+^]_c_ Induces [Ca^2+^]_m_ Overload and Δψm Depolarization in PINK1 KD/KO Cells (A) Arrows mark UV-induced flash photolysis of cells loaded with Ca-NPEGTA, fluo-4, and TMRM. In (A), mouse WT neurons demonstrated an increase in [Ca^2+^]_c_ in response to flash photolysis, with no change in Δψm. (B) In mouse PINK1 KO neurons, flash photolysis induced an increase in [Ca^2+^]_c_ with profound depolarization of the mitochondria. (C) In the same experiment performed on human PINK1 KD neurons, the photolysis-induced rise in [Ca^2+^]_c_ resulted in a dramatic increase in [Ca^2+^]_m_, as demonstrated by the fluo-4 signal in the mitochondrial area (Figure 4Cg). This was rapidly followed by mitochondrial depolarization and subsequent release of fluo-4 from the mitochondrial area. (D) Application of 10 μm CGP-37157 to control neurons induced the same [Ca^2+^]_m_ overload and Δψm depolarization seen in PINK1 KD.

We employed the technique of UV flash photolysis of cells incubated with caged calcium (Ca-NPEGTA) in order to generate a standard calcium signal free from variations in calcium influx/release. UV induced flash photolysis produced a rapid [Ca^2+^]_c_ increase in WT mouse cortical neurons with no associated change in Δψ_m_ (n = 46; Figure 4A). Conversely, the same stimulus in PINK1 KO mouse neurons resulted in profound mitochondrial depolarization (n = 38; Figure 4B). Similarly control human neurons showed a rise in [Ca^2+^]_c_ with no mitochondrial depolarization (n = 35; data not shown), whereas PINK1 KD neurons exhibited a rise in [Ca^2+^]_c_, followed by a stepwise mitochondrial depolarization, where each step coincided with each UV induced [Ca^2+^]_c_ rise (n = 38; Figure 4C). Interestingly, following recovery of the [Ca^2+^]_c_ signal, fluo-4 showed a very bright signal colocalizing completely with the TMRM signal, reflecting high levels of [Ca^2+^]_m_ in PINK1 KD neurons (Figure 4C). Further activation of a calcium signal in these cells caused a profound mitochondrial depolarization and disappearance of the mitochondrially located TMRM signal. Application of FCCP to control neurons caused complete depolarization of the mitochondria. Application of FCCP in PINK1 KD neurons did not produce any further depolarization. Mitochondrial depolarization was prevented by application of CsA. Provision of respiratory chain substrates (5 mM pyruvate and methyl succinate) did not prevent calcium-induced mitochondrial depolarization in PINK1 KO cells.

The sustained increase in [Ca^2+^]_m_ suggested a possible impairment of calcium efflux from the mitochondria, mediated by the Na^+^/Ca^2+^ exchanger. Preincubation of control neurons with a specific inhibitor of the Na^+^/Ca^2+^ exchanger, 10 μM CGP-37157, converted the signal in control neurons to one identical to PINK1 KD neurons mimicking the [Ca^2+^]_m_ overload and inducing mitochondrial depolarization (n = 31; Figure 4D).

In PINK1-deficient cells, physiological [Ca^2+^]_c_ stimuli induce [Ca^2+^]_m_ overload, opening of the PTP, and mitochondrial depolarization.

**Figure 5:**
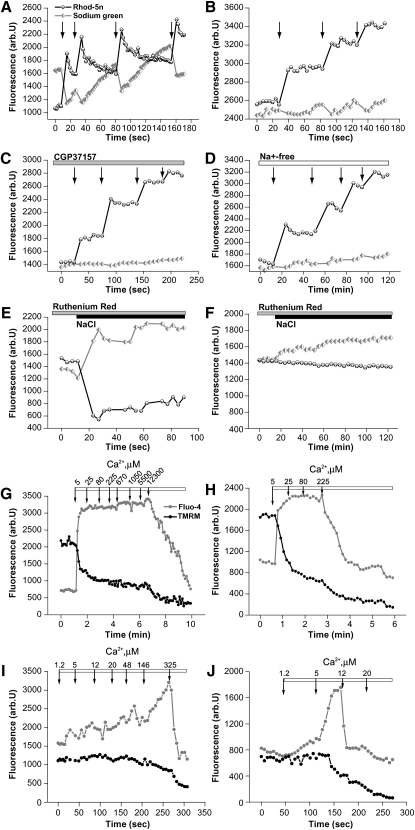
Mitochondrial Calcium Capacity Is Reduced in PINK1 KD/KO Neurons (A–D) Flash photolysis of permeabilized neurons loaded with Sodium Green and Rhod-5n demonstrated a flash-induced increase in [Ca^2+^]_m_ followed by Ca^2+^ efflux and Na^+^ influx in control cells (A). In PINK1 KD cells (B), there was no recovery of the [Ca^2+^]_m_ signal and reduced influx of Na^+^. Application of CGP-37157 (10 μM) to control neurons (C) inhibited the Na^+^/Ca^2+^ exchanger. Removal of Na^+^ from the medium in control neurons (D) also inhibited the Na^+^/Ca^2+^ exchanger. (E and F) Application of 50 mM NaCl in the presence of 7 μM ruthenium red stimulated an increase in Na^+^ and decrease in Ca^2+^ in control mitochondria (E). In contrast, there was minor activation of Na^+^/Ca^2+^ exchange in PINK1 KD mitochondria (F). (G–J) Increasing concentrations of Ca^2+^ were applied to permeabilized human neurons (G and H) or mouse neurons (I and J). Arrows indicate the final free calcium concentration to which mitochondria are exposed. Control human mitochondria (G) demonstrated a much higher Ca^2+^ capacity compared to PINK1 KD mitochondria (H). Control neurons are able to partially maintain the Δψm, until the collapse of the fluo-4 fluorescence. WT mouse neurons (I) also exhibited a higher Ca^2+^ capacity than PINK1 KO neurons (J). Note that human control neurons showed a significantly higher calcium capacity compared to mouse WT neurons.

*Mitochondrial Calcium Transport.* Cells were loaded with Ca-NPEGTA, Sodium Green and Rhod-5n (concentrates in mitochondria), and permeabilized in pseudo-intracellular solution containing 20 μM digitonin (Abramov et al., 2007). In these conditions, a significant fraction of the caged Ca^2+^ is entrapped within mitochondria, enabling selective photolysis-induced release of Ca^2+^ inside the mitochondrial matrix (independent of mitochondrial uptake and Δψ_m_). As the cells are permeabilized, this protocol excludes any difference in intracellular Na^+^ concentration or pH as the basis for altered mitochondrial Ca^2+^ handling. In control neurons, flash photolysis induced a rise in [Ca^2+^]_m_, with a concomitant reduction in the [Na^+^ ]_m_. As demonstrated in Figure 5A, each UV-induced Ca^2+^ peak is followed by Ca^2+^ recovery coinciding with Na^+^ increase, reflecting the activity of the Na^+^/Ca^2+^ exchanger (antiporter). In PINK1 KD neurons each UV-induced Ca^2+^ rise induced a stepwise increase in [Ca^2+^]_m_ without recovery (n = 58), associated with reduced Na^+^ influx compared to control cells (Figure 5B). We were able to inhibit the Na^+^/Ca^2+^ exchanger in control neurons by application of 10 μM CGP-37157 (n = 45 cells; Figure 5C), or by using medium without Na^+^ (n = 63; Figure 5D). In control neurons the Na^+^/Ca^2+^ exchanger could be activated by addition of high (50 mM) Na^+^ to the medium in the presence of 7 μM ruthenium red (inhibitor of Ca^2+^ uniporter), inducing Ca^2+^ efflux from the mitochondria (n = 61; Figure 5E). In PINK1 KD neurons, addition of 50 mM Na^+^ to the medium induced a significantly smaller effect on the [Na^+^]_m_ rise with minimal Ca^2+^ efflux (n = 53 cells; Figure 5F). This data confirms that the Na^+^/Ca^2+^ exchanger is dysfunctional in PINK1 KD neurons.

*Mitochondrial Calcium Capacity.* Permeabilized cells, loaded with TMRM and fluo-4, were exposed to increasing concentrations of exogenous Ca^2+^. Mitochondria are bathed in medium containing pyruvate and malate so the basal Δψm is similar in all cells. We defined Ca^2+^ capacity as the maximum Ca^2+^ concentration tolerated prior to the collapse of Δψm and the rapid disappearance of fluo-4 signal from mitochondria (Murphy et al., 1996; Abramov et al., 2007; Fontaine et al., 1998). In both mouse and human models, we found that the mitochondrial Ca^2+^ uptake capacity of control neurons was dramatically greater than PINK1 KO/KD neurons. The threshold for mPTP opening in WT mouse neurons was 325 μM, n = 25 cells (Figure 5I); for PINK1 KO cells the threshold was only 20 μM, n = 19 (Figure 5J). The calcium capacity could be restored to control values by preincubation of neurons with CsA (0.5 μM) (PINK1 KO mouse neurons, capacity rose from 20 μM to 800 μM, n = 22, data not shown). For control human neurons the threshold was 12.3 mM, n = 22 cells (Figure 5G) compared to 225 μM for PINK1 KD neurons, n = 27 (Figure 5H).

*Basal [Ca^2+^]_m_*. Impaired mitochondrial calcium efflux should result in a higher basal [Ca^2+^]_m_. PINK1 KD cells appeared to have 1.5-fold higher levels of mitochondrial-located Rhod-5N fluorescence (Figures S2Ai and S2Aii) although this is not a ratiometric dye and so cannot be quantitated. We employed an indirect method to measure basal [Ca^2+^]_m_: cells were first treated with an inhibitor of ER calcium ATPase (1 μM thapsigargin) to deplete Ca^2+^ from the ER in Ca^2+^free medium. Application of ionomycin eliminates any remaining [Ca^2+^] gradient between all membranes. Following thapsigargin, the sole remaining gradient is the mitochondria-cytosolic [Ca^2+^] gradient which may be quantified by measurements of releasable Ca^2+^ (Abramov and Duchen, 2003) The response to ionomycin i.e., the [Ca^2+^]_m_ in PINK1 KO mouse neurons and astrocytes was much higher than in control cells (PINK1 KO neurons 196.5 ± 14.5% (n = 69) of WT neurons (n = 88); p < 0.05; PINK1 KO astrocytes 148.6 ± 11.2% (n = 88) of control neurons (n = 74), p < 0.05; Figures S2Bi, S2Bii, and S1C). [Ca^2+^]_m_ was also higher in PINK1 KD human neurons compared to control neurons (PINK1 KD 183.7 ± 11.4% (n = 67) of control (n = 45), p < 0.001; Figure S2D).

### Oxidative Stress

#### Cytosolic ROS

**Figure 6:**
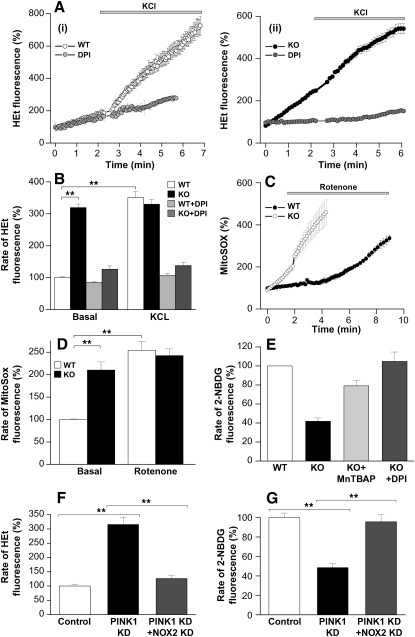
Increase in Mitochondrial and Cytosolic ROS Production in PINK1 KD/KO Neurons (A and B) (A) WT cortical neurons (Ai) demonstrated an increase in HEt fluorescence, and thus cROS production, in response to 50 mM KCl, which was prevented by inhibition of NOX using DPI. PINK1 KO neurons (Aii) exhibited a higher basal level of ROS production than WT neurons. The basal ROS production and the response to KCl were blocked by inhibition of NOX in PINK1 KO neurons. (B) Histogram shows percentage values of rate of HEt fluorescence compared to 100% for WT neurons. (C and D) PINK1 KO neurons displayed a higher basal rate of increase in MitoSOX fluorescence, demonstrating a higher basal production of mROS compared to controls. Inhibition of complex 1 with rotenone induced a significant increase in ROS production in control neurons but only a small increase in ROS production in PINK1 KO neurons. (D) Histogram demonstrates percentage values of rate of MitoSOX fluorescence compared to 100% for WT neurons. (E) The rate of 2-NBDG fluorescence was lower in PINK1 KO neurons compared to WT neurons, reflecting lower glucose uptake. Incubation of PINK1 KO neurons with ROS scavenger MnTBAP increased the glucose uptake. Incubation of PINK1 KO neurons with the NOX inhibitor DPI restored glucose uptake to control levels. (F) The increase in the rate of HEt fluorescence in PINK1 KD cells was abolished by treatment of PINK1 KD cells with NOX-2 siRNA. (G) The reduced rate of 2-NBDG fluorescence in PINK1 KD cells was rescued by treatment of PINK1 KD cells with NOX-2 siRNA. Error bars represent mean ± SEM.

We investigated ROS production in the cytosol and in the mitochondrial matrix by measuring the rate of oxidation of cytosolic hydroethidine (HEt) or the mitochondrially targeted equivalent MitoSOX. The basal cytosolic ROS (cROS) production in PINK1 KO mouse neurons was significantly higher than in WT neurons (PINK1 KO 319.4% ± 11.3% of control, n = 215, p < 0.001; Figures 6Ai and 6Aii). Stimulation of the cells with 50 mM KCl produced a [Ca^2+^]_c_ signal (as shown before) and also stimulated ROS production in control cells (the rate of increase of the HEt fluorescence rose to 351% ± 19.3%, n = 199, p < 0.001). In PINK1 KD/KO cells 50 mM KCl showed a small nonsignificant activation of ROS production (the rate increased from 319.4% ± 11.3% to 330.1% ± 14.7%, n = 215). Both the basal overproduction of ROS, as well as the KCl-stimulated ROS production, was blocked by the NADPH oxidase (NOX) inhibitors DPI (0.5 μM) or AEBCF (20 μM; the basal rate of HEt fluorescence was reduced from 319.4% ± 11.3% to 126.6% ± 10.32%, n = 111, p < 0.001 in DPI-treated cells, Figure 6B). This suggests that cROS production in PINK1 deficiency is due to the basal and Ca^2+^-induced activation of NOX.

To confirm the role of NOX in the production of ROS, we performed siRNA of gp91^phox^ (the major subunit of NOX2 expressed in brain) in human PINK1 KD neuroblastoma cells (n = 2 clones). Reduction of NOX2 expression was confirmed by qRT-PCR (data not shown). Physiologically, application of the main activator of NOX, PMA, further confirmed the presence/absence of the enzyme in ROS production. In NOX2-siRNA PINK1 KD cells, the basal ROS production was significantly reduced compared to PINK1 KD cells treated with scrambled siRNA (from 315.6% ± 25.7% to 126.7% ± 9.4% of control, n = 96 control neuroblastoma cells; n = 92 for PINK1 KD; n = 127 for NOX2-siRNA PINK1 KD; Figure 6F). Thus, in the absence of NOX2, PINK1 KD does not result in cROS overproduction.

*Mitochondrial ROS*. The rate of basal mitochondrial ROS (mROS) production was also significantly higher in PINK1 KD/KO cells than in controls (210.2% ± 18.1% of control, n = 165 for KO neurons; p < 0.001; Figures 6C and 6D). Inhibition of complex I by rotenone (5 μM) stimulated ROS production in control cells but not significantly in PINK1 KD/KO cells (in control neurons the rate of ROS production increased to 253.8% ± 19.9%, n = 187; p < 0.001; in KO neurons the rate rose from 210.2% ± 18.1% to 242.4 ± 14.9%, n = 165). This suggests that mROS production in the PINK1 KD/KO cells is already activated at basal levels by impairment of complex I.

*Consequences of Increased ROS.* PINK1 KO cortical neurons were incubated with the ROS scavenger MnTBAP (10 μM). The resulting reduction of ROS generation in PINK1 KO cells significantly increased the rate of glucose uptake from 41.7% ± 3.9% to 79.1% ± 5.8% of controls, n = 56; p < 0.05 (Figure 6E). Preincubation with an inhibitor of NOX, DPI (0.5 μM), reduced the production of cROS and also restored glucose transport to control values (from 41.7% ± 3.9% to 104.8% ± 9.8%, n = 68; Figure 6E). Furthermore, NOX2-siRNA in PINK1 KD cells also restored glucose uptake to control values (from 48.6% ± 3.9% to 95.6% ± 7.4% of controls, n = 143 for control; n = 131 for PINK1 KD; n = 165 for PINK1 KD with NOX2siRNA; p < 0.001; Figure 6G). However, modulation of ROS production had no effect on the Ca^2^-induced Δψm depolarization (data not shown).

**Figure 7:**
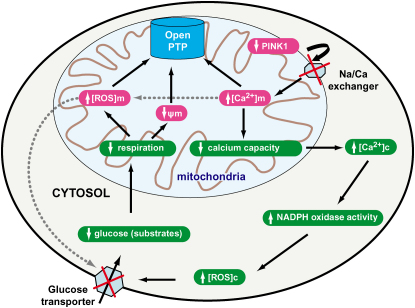
Schematic Diagram Illustrating the Effects of PINK1 within the Mitochondria and Cytosol

Overall, these results suggest that the major consequence of PINK1 deficiency is an increase in [Ca^2+^]_m_, and a reduced Ca^2+^ capacity, secondary to dysfunction of the Na^+^/Ca^2+^ exchanger. Secondary effects of raised [Ca^2+^]_c_, such as activation of NADPH oxidase, cause increased ROS production, which results in impairment of glucose uptake, a reduction in substrate availability, a lower level of respiration, and a lower basal Δψ_m_. These processes and their consequences are outlined in Figure 7.

## Discussion

The first and most robust animal models of Parkinson's disease have been toxin-based models using the complex 1 inhibitors rotenone and MPTP (Betarbet et al., 2000; Forno et al., 1986). Such models recapitulated selective dopaminergic neuronal loss, α-synuclein-positive aggregates, and the clinical features of parkinsonism. However, the use of complex 1 toxins precludes the study of the natural pathophysiological events within mitochondria prior to neuronal death. Genetic models of the autosomal recessive PD have been less successful at reproducing the clinicopathological features of PD in vivo mammalian models (Kitada et al., 2007). However, in vitro models of PINK1 deficiency have produced phenotypes consistent with PD, namely age-related dopaminergic neuronal loss with mitochondrial dysfunction and oxidative stress (Wood-Kaczmar et al., 2008). Moreover, the in vitro models enable the physiologic events occurring within neurons, directly due to a mutation causing PD, to be characterized.

A reduction of complex 1 activity by 30% has been described in brain, muscle, and platelets of patients with sporadic PD (Schapira et al., 1990). Here, we demonstrate that the absence of PINK1 is associated with inhibition of respiration, with concomitant reduced oxygen consumption and an altered redox state. The lowered activity of the respiratory complexes is insufficient to maintain the Δψm, and hence results in a decrease in Δψm. As a result, the mitochondria switch from the production of ATP to the consumption of ATP by the F_1_F_0_-ATPase in order to maintain their Δψm (Campanella et al., 2008). Interestingly, this phenomenon could be reversed by the provision of additional respiratory chain substrates: the increase in respiration in the presence of additional pyruvate resulted in a concomitant switch in the mechanism of Δψm maintenance from hydrolysis of ATP to production of ATP. The ability to reverse the respiratory chain inhibition and repolarize the Δψm has not been previously demonstrated in PD. These data strongly suggest that the respiratory complexes in PINK1 deficiency are intact and that their functional inhibition is in fact secondary to reduced substrate supply. In keeping with other reports in the literature (Scheele et al., 2007), we found that PINK1 loss of function was associated with reduction in glucose uptake at the plasmalemmal membrane in human and mouse neurons. Thus, we conclude that reduced substrate delivery causes the impairment of respiration and reduced Δψm in cells that lack PINK1.

One of the major functions of the mitochondria is the maintenance of calcium homeostasis within the cell. Abnormal calcium homeostasis has been implicated in a range of diseases such as Alzheimer's disease (Abramov et al., 2003, 2004), Huntington's disease, amyotrophic lateral sclerosis, and stroke (Mattson, 2007). In PD, one important observation is that adult dopaminergic neurons are uniquely dependent on calcium channels (rather than sodium channels) to maintain autonomous pacing activity. As a result these neurons are exposed to frequent large influxes of cytosolic calcium, which must be buffered by the mitochondria (Chan et al., 2007). Mitochondrial dysfunction and in particular an inability to handle these calcium loads may render dopaminergic neurons particularly vulnerable to injury. Furthermore, it has been reported that SN and VTA neurons that express higher levels of calcium-binding proteins such as calbindin are spared in both sporadic PD and MPTP toxicity (Damier et al., 1999; Liang et al., 1996). However, the nature of calcium dysregulation and its contribution to neuronal death is largely uncharacterized in PD. We report that human and mouse neurons lacking PINK1 have a higher basal [Ca^2+^]_m_ than control neurons. Furthermore, stimuli that induce a rise in cytosolic calcium cause mitochondrial calcium overload with greatly impaired recovery. As a consequence of this, the mitochondrial calcium uptake capacity is dramatically reduced in the absence of PINK1. Thus, repetitive rises in cytosolic calcium result in mitochondrial calcium overload, leading to premature opening of the PTP, profound mitochondrial depolarization, and ultimately cell death (Crompton, 1999; Nicholls and Budd, 2000). In addition, the opening of the PTP by calcium is modulated by factors such as lowered basal Δψm and raised mROS production (described below), which also occur in PINK1 deficiency.

Accumulation of calcium within the mitochondria matrix depends on both calcium uptake into the mitochondria through an electrogenic uniporter, as well as extrusion of calcium from the mitochondria through Na^+^/Ca^2+^ and H^+^/Ca^2+^ antiporters (Szabadkai et al., 2006). Our data suggest that the cause of mitochondrial calcium accumulation in PINK1 deficiency is a direct impairment of calcium efflux from the mitochondria secondary to dysfunction of the Na^+^/Ca^2+^ exchanger. The reasons for this are (1) following an increase in [Ca^2+^]_m_ there was no mitochondrial efflux of Ca^2+^ and concomitant influx of Na^+^ (which would normally enable recovery of [Ca^2+^]_m_), (2) application of external Na^+^ was able to activate the Na^+^/Ca^2+^ exchanger in control neurons but not in PINK1 KD neurons, and (3) there was no apparent abnormality of calcium influx in PINK1 KD/KO cells, To date, the existence of the calcium uniporter and antiporters has been established only functionally. The Na^+^/Ca^2+^ antiporter was first recognized in heart and brain mitochondria (Carafoli et al., 1974; Crompton et al., 1977, 1978). Although one putative candidate was reported to be isolated from beef heart mitochondria (Li et al., 1992), the proteins that truly account for the activity of these transporters have not yet been identified and confirmed. We have shown physiologically that PINK1 regulates the Na^+^/Ca^2+^exchange mechanism in mitochondria, and that this is fundamental to the role of PINK1 in cell physiology. We are unable to prove biochemically that the Na^+^/Ca^2+^exchanger is a direct substrate or interactor of PINK1 because the molecular identity of this protein remains unclear. In order to fully clarify the relationship between PINK1 and Na^+^/Ca^2+^ exchange, it is necessary to first identify this protein, which is the subject of ongoing research.

One interesting observation in these experiments was the significant difference in the mitochondrial calcium capacity of mouse compared to human neurons. Control mouse neuronal mitochondria depolarized at much lower calcium concentrations than control human neurons. Control mouse neurons did not accumulate mitochondrial calcium in the same way as normal human neurons due to immediate opening of the PTP.

Oxidative stress has long been implicated in sporadic PD: there is evidence of elevated levels of lipid peroxidation markers (4-hydroxynonenal and malondialdehyde) and protein nitration in the substantia nigra and in Lewy bodies of patients with PD (Andersen, 2004). However, it is less clear whether the oxidative stress is causal in PD or a consequence of dysfunctional neurons. At a cellular level, overproduction of ROS would theoretically be able to inhibit the mitochondrial Na^+^/Ca^2+^ exchanger, causing mitochondrial calcium overload, as well as inhibiting the plasmalemmal glucose transporter and reducing respiration (Jornot et al., 1999). In our models, we demonstrate that in the absence of PINK1, there was a significant increase in ROS production from two separate sources. There was an increase in mROS that may be secondary to the increase in mitochondrial calcium, as well as the impairment of respiration. Alternatively, it is recognized that PTP opening per se results in an increase in ROS production through a conformational change in complex I (Batandier et al., 2004). In addition, there was overproduction of cROS, in the form of superoxide by NOX. Increased activity of NOX has been reported to be responsible for the oxidative stress seen in other neurodegenerative diseases and is a major source of ROS production in MPTP-induced cell toxicity (Anantharam et al., 2007). As calcium is known to increase the activation of NOX-2 (Abramov et al., 2005), we postulate that cytosolic calcium may result in an increase in cROS production. Manipulation of ROS production using inhibitors of NOX, NOX-2 (gp91^phox^) siRNA, and ROS scavengers enabled us to distinguish the primary abnormalities from the secondary consequences of ROS production in PINK1 KD/KO neurons. Reduction of ROS production was able to reverse the impaired glucose uptake in PINK1-deficient cells, and thus oxidative stress plays an important role in causing the impaired respiration seen in PINK1 KD/KO. Provision of substrates was also able to overcome the impaired respiration and mitochondrial depolarization. However neither reduction of ROS nor substrate provision was able to affect mitochondrial calcium overload or calcium-induced mitochondrial depolarization in PINK1 KD/KO cells.

In summary, we have characterized the mitochondrial pathophysiology that occurs due to PINK1 loss of function and have attempted to define mechanisms by which PINK1 deficiency renders neurons vulnerable. Within mitochondria there is considerable crosstalk between the bioenergetic function and calcium homeostasis. Our data suggest that as a result of PINK1 deficiency there is primarily an impairment of mitochondrial calcium efflux resulting in mitochondrial calcium overload. This induces a rise in ROS that may further impair calcium efflux and also inhibit glucose uptake, resulting in reduced substrate delivery and impaired respiration. Ultimately, the synergistic action of increased mROS and mitochondrial calcium overload induces opening of the PTP. Opening of the PTP, occurring either as an early event (calcium-induced) or as a late event, has several consequences that exacerbate the mitochondrial pathophysiology and promote cell death: (1) PTP opening will further increase ROS production in the mitochondria, (2) PTP opening will result in reduced rate of maximal respiration through pyridine nucleotide depletion (Di Lisa et al., 2001), and (3) PTP opening will cause cytochrome *c* release and neuronal apoptosis. Neurons of the substantia nigra, where there is increased oxidative stress and large calcium influxes, will be particularly susceptible to mitochondrial apoptosis via these mechanisms.

## Experimental Procedures

### Cell Models

The generation, characterization, and maintenance of the neuroblastoma, human neuron, and mouse neuronal cultures are described briefly in the Supplemental Experimental Procedures and have been published elsewhere (Wood-Kaczmar et al., 2008).

### Loading of Cells

For measurements of [Ca^2+^]_c_ and [Ca^2+^]_m_, cells were loaded for 30 min at room temperature with 5 μM of either fura-2 AM or fluo-4 AM in combination with 5 μM of either X-Rhod-1 AM or Rhod-5n, and 0.005% pluronic acid in a HEPES-buffered salt solution (HBSS) composed of 156 mM NaCl, 3 mM KCl, 2 mM MgSO_4_, 1.25 mM KH_2_PO_4_, 2 mM CaCl_2_, 10 mM glucose, and 10 mM HEPES (pH adjusted to 7.35 with NaOH). For flash photolysis experiments, caged Ca^2+^, 10 μM *o*-nitrophenyl EGTA, AM (NP-EGTA, AM) was loaded at the same time as the other indicators, and Ca^2+^-free medium containing 0.5 mM EGTA was used. For experiments using permeabilized cells, neurons were exposed to 20 μM digitonin in a “pseudo-intracellular” solution consisting of 135 mM KCl, 10 mM NaCl, 20 mM HEPES, 5 mM pyruvate, 5 mM malate, 0.5 mM KH_2_PO_4_, 1 mM MgCl_2_, 5 mM EGTA, and 1.86 mM CaCl_2_ (to yield a free [Ca^2+^] of ∼100 nM).

For measurements of Δψ_m_, cells were loaded with 25 nM tetramethylrodamine methylester (TMRM) for 30 min at room temperature, and the dye was present during the experiment. TMRM is used in the redistribution mode to assess Δψm, and therefore a reduction in TMRM fluorescence represents Δψm depolarization. Alternatively, Rh123 (26 μM, equivalent to 10 μg ml^−1^; Molecular Probes) was added to cells during the last 15 min and washed prior to the experiment. Under these loading conditions, Rh123 is nontoxic and gives a reliable and reproducible measure of Δψ_m_ through the “dequench” of mitochondrial fluorescence, and thus an increase in the Rh123 signal reflects mitochondrial depolarization.

For measurement of mROS production, cells were preincubated with MitoSOX (5 μM, Molecular Probes, Eugene, OR) for 10 min at room temperature. For measurement of cROS production, dihydroethidium (2 μM) was present in the solution during the experiment. No preincubation (“loading”) was used for dihydroethidium to limit the intracellular accumulation of oxidized products.

For glucose uptake experiments, the uptake of the fluorescent glucose homolog 2-NBDG (Invitrogen) was measured by the addition of 40 nM 2-NBDG in the presence of 0.25 mM glucose during a period of continuous imaging.

### Fluorescence Measurements

Fluorescence measurements were obtained on an epifluorescence inverted microscope equipped with a ×20 fluorite objective. [Ca^2+^]_i_ and ΔΨ_m_ were monitored in single cells using excitation light provided by a Xenon arc lamp, the beam passing sequentially through 10 nm band pass filters centered at 340, 380, and 490 nm housed in computer-controlled filter wheel (Cairn Research, Kent, UK). Emitted fluorescence light was reflected through a 515 nm long-pass filter to a cooled CCD camera (Hamamatsu, Orca ER) and digitized to 12 bit resolution (Digital Pixel Ltd, UK). All imaging data were collected and analyzed using software from Andor (Belfast, UK). Wherever possible, we have normalized the signals between resting level (set to 0) and a maximal signal generated in response to the uncoupler FCCP (1 μM; set to 100%).

Confocal images were obtained using a Zeiss 510 uv-vis CLSM equipped with a META detection system and a 40× oil immersion objective. The 488 nm Argon laser line was used to excite fluo-4 fluorescence, which was measured using a band-pass filter from 505 to 550 nm. Illumination intensity was kept to a minimum (at 0.1%–0.2% of laser output) to avoid phototoxicity, and the pinhole was set to give an optical slice of ∼2 μm. TMRM was excited using the 543 nm laser line and fluorescence measured using a 560 nm long-pass filter. For HEt, MitoSOX, X-Rhod, or Rhod-5n measurements the 543 nm laser line and 560 nm long-pass filter were used. NADH autofluorescence was excited at 351 and measured at 375–470 nm. 2-NDBG was excited at 458 nm, and fluorescence was measured at 520 nm. All data presented were obtained from at least five coverslips and two to three different cell preparations.

### Oxygen Respiratory Activity

To measure respiration rate in intact cells, approximately 1 × 10^7^cells were suspended in respiration medium (HBSS) with 10 mM D-glucose in a Clark-type oxygen electrode thermostatically maintained at 37°C. The oxygen electrode was calibrated with air-saturated water, assuming 406 nmol O atoms/ml at 37°C. Oxygen consumption was measured over 10 min with addition of oligomycin (final concentration 2 μg/ml) and 0.5 μM FCCP. All data were obtained using a MacLab system with Chart recording software.

### Statistical Analysis

Data were generated from a minimum of three independent experiments, using a minimum of 20 cells per experiment and replication in two to three different clones for PINK1 KD. Statistical analysis and exponential curve fitting were performed using Origin 7 (Microcal Software Inc., Northampton, MA) software. Data were analyzed by parametric Student's t tests and significance expressed: ^∗^p < 0.05, ^∗∗^p < 0.005. For all graphs, error bars represent mean ± SEM.

## Supplemental Data

Document S1. Supplemental Experimental Procedures and Two Figures
