# Supplementary material for: PINK1-Associated Parkinson's Disease Is Caused by Neuronal Vulnerability to Calcium-Induced Cell Death
Source: Mol Cell. 2009 Mar 13;33(5-3):627–38. doi: 10.1016/j.molcel.2009.02.013 (PMC2724101; doi:10.1016/j.molcel.2009.02.013)

Molecular Cell, Volume *33*

Supplemental Data

PINK1-Associated Parkinson's Disease Is Caused by Neuronal Vulnerability to Calcium-Induced Cell Death

Sonia Gandhi, Alison Wood-Kaczmar, Zhi Yao, Helene Plun-Favreau, Emma Deas, Kristina Klupsch, Julian Downward, David S. Latchman, Sarah J. Tabrizi, Nicholas W. Wood, Michael R. Duchen, and Andrey Y. Abramov

Supplemental Experimental Procedures

Cell culture: human PINK1 KD cell models

ReNcell VM (ReNcell VM) neural stem cells (NSCs) were derived from 9 week human fetal ventral mesencephalon as described. NSCs were grown in laminin coated flasks/plates (Trevigen; 20 µg/ml in DMEM:F-12) in B27 medium (DMEM:F-12 with 1X B27 supplement, 2mM Glutamine, 10 Units/ml heparin and 50 mg/ml gentamycin) containing growth factors human bFGF [10ng/ml, Peprotech] and human EGF [20ng/ml, Peprotech]. Differentiation to human neurons was performed using a standard method. SH-SY5Y human neuroblastoma cells were maintained as described (Muqit et al., 2006). Generation of stable PINK1 KD in SH-SY5Y cells and NSCs has been previously described. Briefly mammalian expression vectors expressing shRNA targeted to PINK1 mRNA sequence were packaged into retroviral particles, and used to infect SH-SY5Y cells and NSCs. Stable cell lines expressing PINK1 shRNA were screened for PINK1 expression using semi-quantitative real time PCR. 3 clones of each cell type with > 90% reduction in gene expression were selected for use in all experiments. 3 clones expressing empty vector were used as controls. For PINK1 rescue experiments, 3 silent mutations were introduced in the wild-type PINK1 sequence (pcDNA 3.1 vector) in the region targeted by the shRNA. PINK1 KD SHSY5Y cells were then transfected with wild-type PINK1-pcDNA 3.1, or K219M-PINK1-pcDNA3.1 and double stable cell lines were generated. RT-PCR confirmed re-expression of PINK1 in these cell lines.

Cell culture: primary PINK1 KO mouse neuronal model

The PINK1 deficient mice were generated by Lexicon Genetics Inc. (The Woodlands, Texas, USA). For primary mouse cortical cultures, embryos were taken at gestational stage E16-E17. For primary mouse midbrain cultures pups were taken at postnatal D4-6. Embryos/pups were obtained either by crossing homozygote animals and comparing same age control animals, or by crossing two heterozygote animals and comparing genotypes within a litter. Animal husbandry and experimental procedures were performed in full compliance with the United Kingdom

Animal (Scientific Procedures) Act of 1986. Heads were collected in chilled dissection medium [HBSS without calcium or magnesium, supplemented with 0.45% (v/v) D-(+) glucose, 1 mM sodium pyruvate and 10 mM HEPES pH 7.4]. The whole brain was removed from the skull case in a Petri dish containing chilled dissection medium under sterile conditions, and the meninges were removed. The cortices or midbrain was carefully dissected and transferred to a sterile micro tube containing ~0.5 ml chilled dissection medium and allowed to settle under gravity. Dissection medium was replaced with 500 μl pre-warmed trypsin solution for 15 minutes at 37˚C with gentle agitation halfway through incubation. The trypsination solution was aspirated and the cortices/midbrain washed three times with 500 μl pre-warmed attachment medium [1x Modified Eagles Medium (MEM) with Earles and glutamine (Invitrogen), 1 mM pyruvic acid, 0.45% (w/v) D-(+) glucose (Sigma) and 10% (v/v) heat inactivated fetal bovine serum (Invitrogen)]. The tissue was triturated using sterile fire-polished glass pipettes of differing pore diameter until a smooth suspension was produced. Cells were placed in a humidified CO_2 incubator (5% CO_2 in air) at 37°C for 3-4 hrs, before replacing the attachment medium with prewarmed maintenance medium [Neurobasal medium (Invitrogen), 2% (v/v) B27 supplement, 2 mM glutamine, 100 I.U./ml penicillin and 100 I.U./ml streptomycin (Sigma) and 0.45% (v/v) D-(+) glucose]. Once the cells were in maintenance medium, half of the medium was replaced weekly. All live cell imaging experiments were performed between d10-d14 in culture.

Figure S1. PINK1 deficiency causes reduced glucose uptake in neuroblastoma cells and mammalian neurons

(A) Measurement of fluorescent 2-NBDG revealed that the rate of glucose uptake in PINK1 KO cortical mouse neurons was 41.7±3.9% of WT neurons.
(B) PINK1 KD neuroblastoma cells exhibited a reduction in glucose uptake: 63.8±5.4% of control cells.

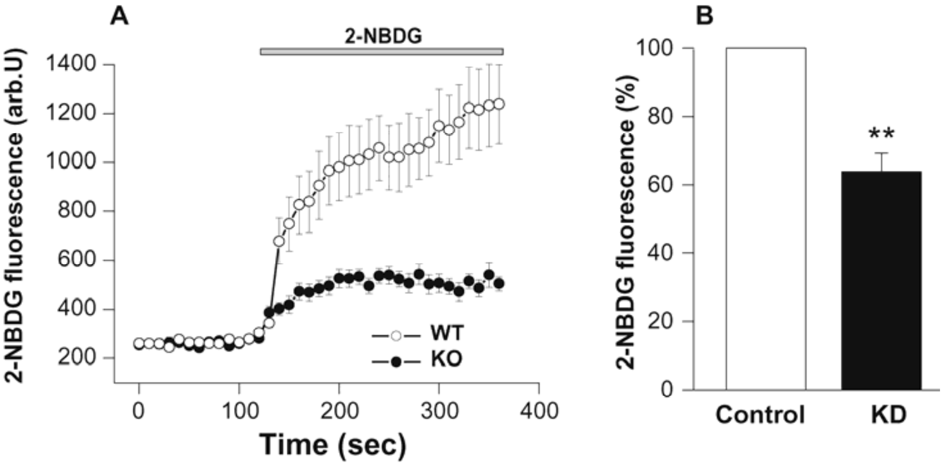

Figure S2. PINK1 KO neurons have an increased $[Ca^{2++}]_m$ compared to wild-type neurons

(A) Basal mitochondrial Rhod-5N fluorescence signal is higher in PINK1 KD neurons (Aii) compared to control human neurons (Ai)
(B) Differences in response to 5µM ionomycin in thapsigargin (0.5 µM) treated cells in calcium free medium (0.5mM EGTA). PINK1 KO neurons and astrocytes (Bii) showed a significantly higher change in $[Ca^{2+}]_c$ following ionomycin treatment, than wild-type cells (Bi) reflecting a higher $[Ca^{2+}]_m$ in PINK1 KO neurons.
(C) Histogram demonstrating %age difference in $[Ca^{2+}]_m$ between PINK1 KO mouse neurons and astrocytes and controls.
(D) demonstrates a similar increase in $[Ca^{2+}]_m$ for human PINK1 KD neurons compared to control neurons.

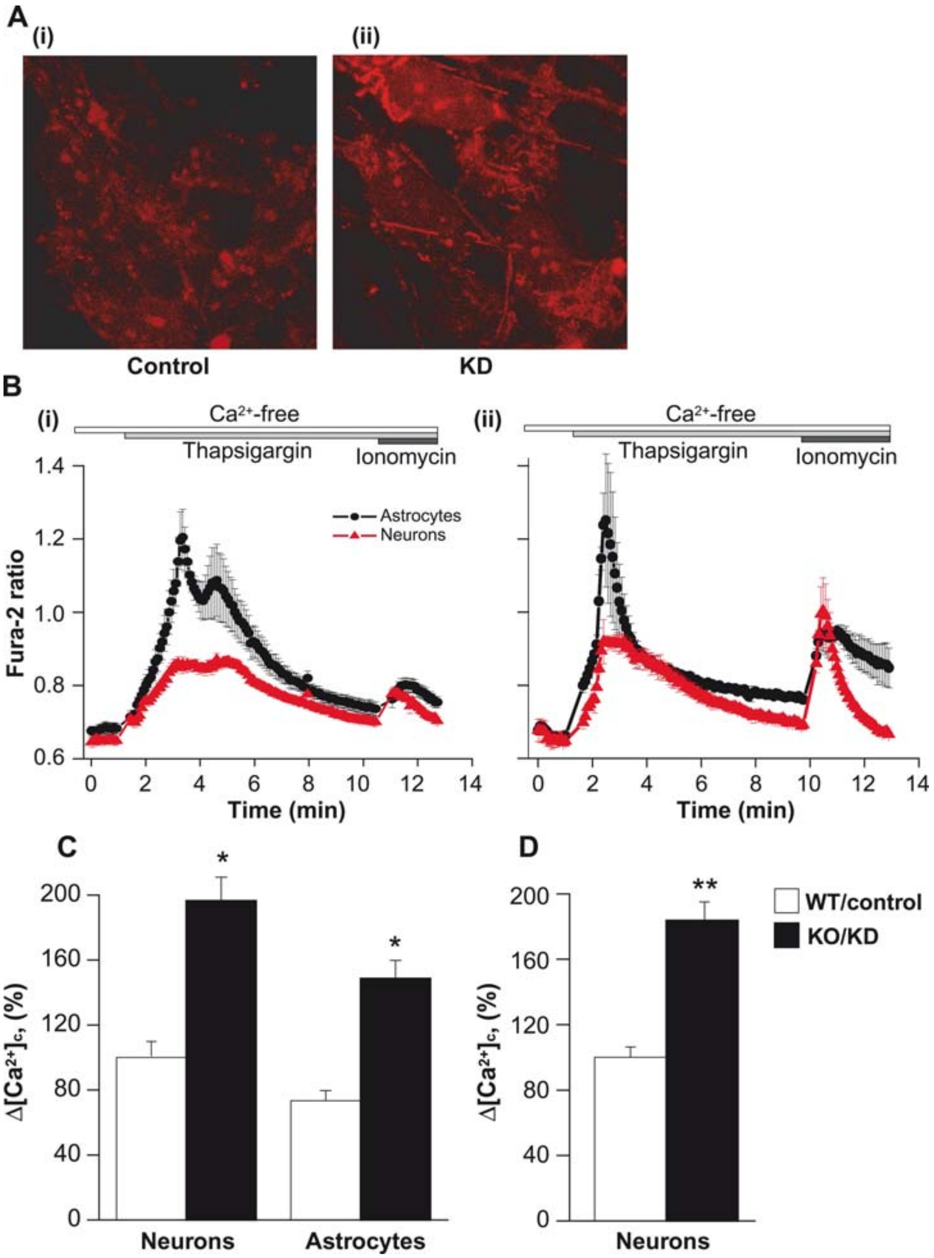

Supplement: Document S1. Supplemental Experimental Procedures and Two Figures [file mmc1.pdf]
